# Neutralization of Botulinum Neurotoxin by a Human Monoclonal Antibody Specific for the Catalytic Light Chain

**DOI:** 10.1371/journal.pone.0003023

**Published:** 2008-08-20

**Authors:** Sharad P. Adekar, Tsuyoshi Takahashi, R. Mark Jones, Fetweh H. Al-Saleem, Denise M. Ancharski, Michael J. Root, B. P. Kapadnis, Lance L. Simpson, Scott K. Dessain

**Affiliations:** 1 Lankenau Institute for Medical Research, Wynnewood, Pennsylvania, United States of America; 2 Division of Infectious Diseases and Environmental Medicine, Thomas Jefferson University, Philadelphia, Pennsylvania, United States of America; 3 Department of Biochemistry and Molecular Biology, Kimmel Cancer Center, Thomas Jefferson University, Philadelphia, Pennsylvania, United States of America; 4 Department of Microbiology, University of Pune, Pune, India; Centre de Recherche Public-Santé, Luxembourg

## Abstract

**Background:**

Botulinum neurotoxins (BoNT) are a family of category A select bioterror agents and the most potent biological toxins known. Cloned antibody therapeutics hold considerable promise as BoNT therapeutics, but the therapeutic utility of antibodies that bind the BoNT light chain domain (LC), a metalloprotease that functions in the cytosol of cholinergic neurons, has not been thoroughly explored.

**Methods and Findings:**

We used an optimized hybridoma method to clone a fully human antibody specific for the LC of serotype A BoNT (BoNT/A). The 4LCA antibody demonstrated potent *in vivo* neutralization when administered alone and collaborated with an antibody specific for the HC. In Neuro-2a neuroblastoma cells, the 4LCA antibody prevented the cleavage of the BoNT/A proteolytic target, SNAP-25. Unlike an antibody specific for the HC, the 4LCA antibody did not block entry of BoNT/A into cultured cells. Instead, it was taken up into synaptic vesicles along with BoNT/A. The 4LCA antibody also directly inhibited BoNT/A catalytic activity *in vitro*.

**Conclusions:**

An antibody specific for the BoNT/A LC can potently inhibit BoNT/A *in vivo* and *in vitro*, using mechanisms not previously associated with BoNT-neutralizing antibodies. Antibodies specific for BoNT LC may be valuable components of an antibody antidote for BoNT exposure.

## Introduction

Botulinum neurotoxins (BoNTs) are a family of category A select bioterror agents and the most potent biological toxins known [1]. BoNTs are produced by bacteria of the genus *Clostridium* and are the cause of the paralytic disease, botulism. BoNT exposure can occur either by respiratory or gastrointestinal routes. Clinically, exposure to BoNT results in a flaccid peripheral and bulbar paralysis that can require weeks to months of ventilatory and intensive care unit support. BoNT has been prepared for use as a bioweapon by governments as well as a terrorist organization. An estimate of the possible effects of an intentional environmental release of BoNT predicted 10% incapacitation or death for those within 0.5 km down-wind of the release site [1]. In addition, the U.S. milk supply may be particularly vulnerable to a terrorist attack with BoNT [2].

BoNTs exist in seven serotypes (A–G), each of which has distinct antigenic and functional attributes. However, every BoNT is a heteromeric molecule that consists of a 100 kD heavy chain domain (HC) and a 50 kD light chain domain (LC). The steps of BoNT intoxication have been well defined [3]. The HC portion of the toxin mediates binding to cholinergic nerve synapses. BoNT binding to neurons involves recognition of low affinity ganglioside binding sites as well as high affinity protein binding sites, such as SV2, the synaptic vesicle protein recognized by serotype A BoNT (BoNT/A) [4], [5]. Once bound, the toxin enters the neurons by endocytosis. This is followed by acidification of the endosomes, which induces translocation of the LC into the cytosol, in a process that is facilitated by the HC [3]. In the cytosol, the LC domains use a zinc metalloprotease activity to cleave components of the SNARE (soluble N-ethylmaleimide-sensitive factor attachment protein receptor) complex, a set of proteins required for synaptic vesicle fusion and the release of the neurotransmitter acetylcholine. One of the SNARE proteins, the synaptosomal-associated 25 kDa protein (SNAP-25), is specifically cleaved and inactivated by the BoNT/A LC, which removes a 9-amino acid C-terminal peptide [6]. As a consequence, acetylcholine cannot be released into the neuromuscular synapse and paralysis results.

Immunotherapy is presently considered to be the most effective immediate response to BoNT exposure, but the human anti-BoNT antiserum (BabyBIG) is in very limited supply and equine antisera can induce serum sickness and anaphylaxis [1], [7]. Monoclonal antibodies may be a viable substitute for polyclonal antisera [8], [9]. An important principle is that combinations of antibodies synergistically cooperate in neutralization potency [10]. Kinetic studies have shown that a BoNT/A-specific triplex antibody combination exhibits cooperative binding to the toxin, increasing the stability of the antibody∶toxin complex [10]. Epitope mapping has shown that the three antibodies together cover a large region of the surface of the BoNT/A HC domain required for neuron binding [11]. In addition, pharmacokinetic studies have demonstrated that immune complexes formed in the blood circulation between BoNT and polyclonal antisera rapidly sequester the toxin in the liver and spleen [12].

A majority of the effort to create combinations of antibodies for use as BoNT therapeutics has concentrated on antibodies that bind the HC. These antibodies can potentially inhibit the interaction of BoNT with its neuron receptors [8], [13]. We explored the potential for an antibody directed at the LC to neutralize toxin *in vitro* and *in vivo*. We recently described a novel hybridoma method for cloning human antibodies [14], [15]. We have used this method to clone a fully human antibody specific for the BoNT/A LC. We have found that it has novel features that enable it to potently neutralize BoNT/A *in vivo* and *in vitro*.

## Results

### A human antibody specific for the botulinum neurotoxin serotype A light chain catalytic domain

We obtained peripheral blood from a volunteer on the 8^th^ day following a fifth dose of the pentavalent botulinum toxoid vaccine. The mononuclear cell population was purified by Ficoll gradient centrifugation and cells expressing the CD27 antigen were isolated by magnetic beads conjugated to an anti-CD27 antibody. The selected cells were cultured for 8 days over a monolayer of irradiated tCD40L fibroblast cells, which express the human CD40 ligand, in culture medium that contained interleukin-4, interleukin-10, and cyclosporine [14], [16]. The cultured cells were fused to the B5-6T cell line, a human-murine hybrid myeloma cell that expresses the hTERT and mIL-6 genes [17]. After HAT selection, a hybridoma expressing an antibody reactive with bacterially expressed BoNT/A light chain was identified and subcloned by limiting dilution three times.

**Figure 1:**
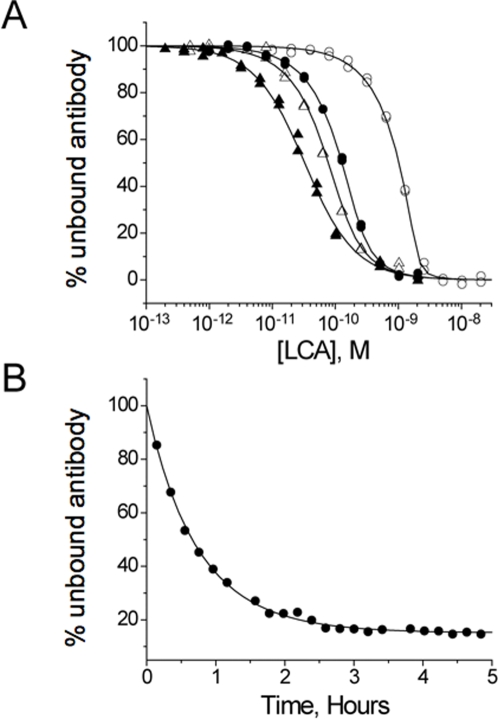
Kinetic exclusion analysis (KinExA) of the solution binding properties of the 4LCA antibody and BoNT/A LC. (A) The equilibrium binding affinity was determined by titrating recombinant BoNT/A LC into solutions of fixed 4LCA concentration. At equilibrium, free 4LCA was captured by passage through a bead matrix conjugated to LC and measured by binding of a fluorescent secondary antibody. The data were fit using the manufacturer's software to a bimolecular binding model, giving an optimized KD value of 31±5×10^−12^ M. (B) The association rate constant (k_on_) was measured by mixing 4LCA (200 pM) with LC (10 pM) and measuring the concentration of free antibody concentration over time. The average k_on_ value for 4LCA was estimated to be 2.3±0.1×10^6^ M^−1^ s^−1^, which gives a calculated k_off_ value of 7.1×10^−5^ s^−1^.

By ELISA, the 4LCA antibody binds specifically to the BoNT/A LC, and not to the BoNT/A heavy chain (data not shown). We used the kinetic exclusion assay (KinExA) to measure the binding affinity of the 4LCA antibody for the BoNT/A LC (Figure 1). Equilibrium binding analysis measured a K_D_ value of 31±5×10^−12^ M, with a k_on_ value of 2.3±0.1×10^6^ M^−1^ s^−1^. The calculated k_off_ value of 7.1×10^−5^ s^−1^ estimates an average bound lifetime of a 4LCA/LC complex to be ∼4 hours at room temperature.

The 4LCA antibody is comprised of an IgG1 heavy chain and a lambda light chain (data not shown). We obtained the sequence of the heavy chain and light chain variable domains of the 4LCA antibody, using RT-PCR with consensus primers to amplify these domains (Table 1) [14]. The heavy chain is derived from the V4-39*01 gene, with 16 mutations in its variable domain, and the light chain uses the V3-21*01 gene, with 11 mutations. These are consistent with an origin of the antibody within a CD27+, post-germinal center B-cell.

**Table 1:** 4LCA variable domain usage, CDR3 sequences, and mutation status.

	V-gene	J-gene	D-gene	CDR3 sequence	CDR3 length	Muts
IgG HC	V4-39*01	J5*02	D3-16*01	ATFLGRGAPFDP	12	20
IgG LC	V3-21*01	J3*02	N/A	QVWGSGSDHPGV	12	11

CDR3, complementary determining region 3. Muts, mutations in the entire IgG HC and LC variable domains.

### Neutralizing activity of the 4LCA antibody *in vivo*

We tested the ability of 4LCA to neutralize BoNT in the standard mouse protection assay. We incubated 100 µg 4LCA with BoNT/A for 1 hour prior to intravenous injection into Swiss Webster mice, testing groups of 6 mice at each dose level. Mice were observed for morbidity and mortality over 30 days. Complete protection was observed with doses up to 25 LD50s, which exceeds by 10-fold the 2.5 LD50s that we had previously shown could be neutralized by the 6A antibody, a human IgG specific for the BoNT/A heavy chain (Table 2) [14]. Partial protection, as indicated by increased survival compared to antibody-free control mice, was afforded with higher doses (Table 2). At 100 LD50s, mice receiving 4LCA survived 85 hours, compared to 8 hours for the control mice, and at 250 LD50, 4LCA mice survived 40 hours, compared to 4 hours for the control mice. As combinations of monoclonal antibodies have demonstrated synergy in BoNT/A neutralization [8], we next tested for synergy with the 6A antibody. We mixed 50 µg each of the 4LCA and 6A antibodies (total of 100 µg) with 1,000 LD50s BoNT/A and tested the combination by intravenous injection. In two separate experiments, all of the mice that received the antibodies survived, in contrast to the control mice, which died in less than 3 hours.

**Table 2:** BoNT neutralization activity of the 4LCA human monoclonal antibody in the mouse protection assay.

huMAb	BoNT/A LD_50_	Survival time hours	Survival alive/tested
Control	25	8	0/6
4LCA	25	*	**6/6**
6A	25	24	0/6
Control	100	<8	0/6
4LCA	100	85	0/6
Control	250	<4	0/6
4LCA	250	40	0/6
Control	1000	<3	0/6
4LCA+6A	1000	*	**6/6**

* mice were healthy at 30 days.

### Neutralization of BoNT activity by the 4LCA antibody

**Figure 2:**
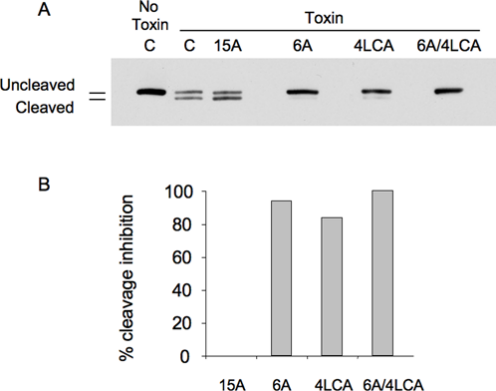
Effect of human antibodies on cleavage of SNAP-25 by BoNT/A in Neuro-2a cells. Neuro-2a cells were incubated with BoNT/A for 48 hours in the presence or absence of human antibody. (A) Cleavage of SNAP-25 was detected by immunoblotting of whole cell extracts. Removal of a 9 amino acid C-terminal fragment gives a band of reduced size (Cleaved) in addition to an intact SNAP-25 band (Uncleaved). Cells received either the antibodies indicated or culture medium without antibody (M). (B) The extent of SNAP-25 cleavage in the experiment shown in Figure 2A was quantified by scanning densitometry. Values are reported normalized to the cell sample that contained BoNT/A but no antibody.

We analyzed the neutralization activity of the 4LCA antibody using cell culture and enzymatic assays. In the first assay, we tested the ability of the 4LCA to inhibit cleavage of SNAP-25, the proteolytic substrate of the BoNT/A LC, in Neuro-2a murine neuroblastoma cells [18], [19]. Cleavage of SNAP-25 by the BoNT/A LC removes 9 C-terminal amino acids, resulting in a truncated form of SNAP-25 that can be detected by immunoblotting. We compared the 4LCA and 6A antibodies with the 15A antibody, which binds BoNT/A with low affinity and has no neutralizing activity *in vivo* (data not shown). We incubated 2 µg of BoNT/A with 500 µg of human monoclonal antibody and then applied the mixtures to Neuro-2a cell monolayers. After 48 hours, whole-cell extracts were assayed by immunoblotting with an antibody specific for SNAP-25. As shown in Figure 2a, exposure of the cells to BoNT/A alone resulted in the appearance of the proteolytic cleavage product. The 15A (non-neutralizing) antibody had no effect. In contrast, the 4LCA and 6A antibodies inhibited 92% and 84% of the cleavage induced by BoNT/A, respectively (Figure 2a and 2b). When administered together, the 4LCA and 6A had a combined effect that completely inhibited SNAP-25 cleavage (Figure 2a and 2b). Thus, the *in vivo* neutralizing activity of the 4LCA and 6A antibodies is correlated with the ability of the antibodies to inhibit SNAP-25 cleavage in a cultured cell line.

**Figure 3:**
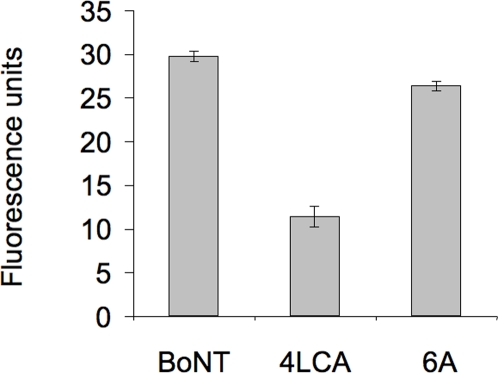
Inhibition of the catalytic activity of the BoNT/A metalloprotease by the 4LCA antibody *in vitro*. In this fluorescence resonance energy transfer assay (FRET), cleavage of the SNAPtide by BoNT/A LC un-quenches FITC fluorescence, which is quantified as arbitrary fluorescence units. In triplicate samples, the SNAPtide was incubated with BoNT/A alone or with the 6A or 4LCA antibodies and fluorescence was measured. Standard deviation (s.d.) is indicated by error bars.

Because the 4LCA directly binds to the BoNT/A LC, we assessed whether it can inhibit the catalytic function of the LC. We used a fluorescence resonance energy transfer assay (FRET), which measures cleavage of a synthetic SNAP-25 peptide (SNAPtide) [20]. The SNAPtide is conjugated to FITC and DABCYL, a molecule that quenches the fluorescent FITC emission. Cleavage of the peptide un-quenches the FITC signal, which can then be measured as an indicator of metalloprotease activity. We incubated the 4LCA and 6A antibodies with the SNAPtide in triplicate reactions and measured the FITC fluorescence intensity (Figure 3). The 6A antibody did not interfere with cleavage of the SNAPtide, giving 26 fluorescence units (FIU), compared to 30 FIU for the control, whereas the 4LCA antibody inhibited SNAPtide cleavage, giving 11 FIU. Thus, binding of the 4LCA antibody can directly inhibit BoNT/A metalloprotease activity.

### BoNT/A-dependent internalization of the 4LCA antibody by Neuro-2a cells

**Figure 4:**
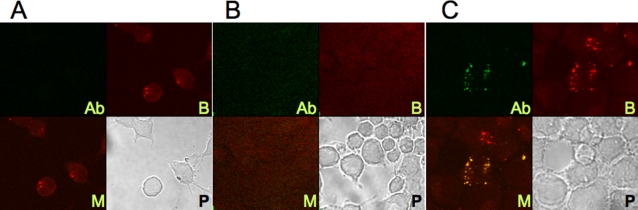
The effect of BoNT/A-specific antibodies on endocytosis of BoNT/A by Neuro-2a cells. Neuro-2a cells were cultured with fluorescently-labeled BoNT/A (red), with or without human antibody (green), and visualized by confocal microscopy. Panel (A) No antibody. (B) 6A antibody. (C) 4LCA antibody. In each panel, 4 images of each field are shown: BoNT/A (BT), Antibody (AB), merged (M), and phase contrast (P).

BoNT-specific antibodies with neutralization capability may directly impair the interaction of toxin with its target cells [11]. We tested the effect of the 4LCA and 6A antibodies on the interaction of BoNT/A with Neuro-2a cells. We labeled the 4LCA and 6A antibodies with DyLight 549 and BoNT/A with DyLight 488 and examined the cells using confocal microscopy (Figure 4). BoNT/A was taken up by the Neuro-2a cells in a punctate pattern consistent with internalization within endocytosed synaptic vesicles [21] (Figure 4A). When BoNT/A was co-incubated with the 6A antibody, uptake of BoNT/A by the Neuro-2a cells was completely prevented (Figure 4B), consistent with the specificity of the 6A antibody for the BoNT/A 50 kD C-terminal, neuron-binding domain [14]. The 4LCA antibody did not prevent internalization of the BoNT/A. Instead, it appeared to enter the cells, giving signals that overlapped with the labeled BoNT/A (Figure 4C). No 4LCA signals were observed that did not co-localize with BoNT/A signals, even though the 4LCA was in 500-fold molar excess compared to BoNT/A. This indicates that the entry of the 4LCA into the Neuro-2a cells was dependent on its binding to BoNT/A.

## Discussion

In the present study, we cloned and characterized the first fully human monoclonal antibody specific for the BoNT/A LC. The 4LCA antibody is an IgG1 λ antibody with picomolar binding affinity. It demonstrated a potent neutralizing activity *in vivo*, with 10-fold more activity than the 6A antibody, which binds the BoNT/A HC and is the most potent fully human BoNT/A neutralizing antibody previously described. An equimolar combination of the 6A and 4LCA antibodies was able to neutralize BoNT/A with a potency of 10,000 LD50/mg antibody. This potency is over three times greater than the BoNT/A neutralization standard devised for the commercially available BabyBig human anti-BoNT antiserum (3,000 LD50/mg antibody) [22]. These results indicate that the antibodies that bind the BoNT/A LC can have significant neutralizing abilities and should be useful components of oligoclonal antibody therapeutics for BoNT exposures.

As would be expected with a neutralizing antibody specific for the HC, the 6A antibody completely inhibited endocytosis of the toxin by Neuro 2a cells. In contrast, the 4LCA antibody was endocytosed along with BoNT/A and co-localized with the BoNT within intracellular vesicles. Thus, the 4LCA appears to inhibit toxin function at the final stages of the intoxication process, after BoNT enters the cell and before it cleaves its intracellular substrate, SNAP-25. The ability of an antibody to neutralize BoNT at an intracellular level has not previously been described. A number of mechanisms may account for this activity, but it is most likely that the antibody causes the LC to be retained within endosomes. Fisher and Montal observed that an LC-specific Fab antibody prevented translocation of the BoNT/A LC across a model endosomal plasma membrane [23]. In such a case, the 4LCA antibody may force the return of the BoNT to the cell surface through synaptic vesicle recycling [21]. A similar phenomenon was observed with the A subunit of the Shiga Toxin 2 (Stx2), which normally undergoes transit from early endosomes to the Golgi and endoplasmic reticulum. Binding of Stx2 to a neutralizing antibody caused recycling of the bound toxin back to the cell membrane [24].

The 4LCA antibody was also able to inhibit the catalytic activity of BoNT/A in an *in vitro* assay with a synthetic substrate. This capability is not a feature common to all LCA-specific antibodies, since a polyclonal rabbit anti-BoNT/A antiserum, immunoreactive with the BoNT/A LC, did not affect the cleavage of the SNAPtide (Supplemental Figure S2 in [25]). In its native state, BoNT/A contains a “translocation domain belt”, which occupies much of the SNAP-25 binding and catalytic site of the LC domain [26]. Binding of the 4LCA antibody to the holotoxin is apparently not inhibited by the presence of the translocation domain belt, as it binds the BoNT/A holotoxin with approximately the same affinity as it does to the recombinant LC (data not shown). This suggests that the 4LCA antibody binds the LC at a site independent of the SNAP-25 substrate binding site and therefore may inhibit the LC by a non-competitive mechanism. Because the antibody-bound BoNT appears to be trapped in endocytic vesicles, it is not yet clear whether the ability of the 4LCA antibody to inhibit LC catalytic function can contribute to its neutralization potency *in vivo*.

A theoretical advantage to the inclusion of a light chain-specific antibody in an oligoclonal antibody therapeutic is that it provides an additional layer of protection from BoNT, intracellular neutralization, at the same time as it contributes to clearance of the toxin by immune complex-mediated mechanisms. Because a BoNT/A therapeutic cannot be thoroughly tested in humans in a non-emergency setting, it will be important to increase the confidence in the efficacy of a BoNT/A antitoxin by incorporating antibodies with as many complementary neutralizing functions as possible. In this context, the light chain is an important target because it offers an ability to inhibit a BoNT/A molecule that has evaded neutralization at the level of toxin uptake, bloodstream circulation, and neuron binding.

A wide variety of bacterial toxins have catalytic domains that enter cells through the aid of heteromeric sub-units [27]. Because the 4LCA antibody was cloned from a human subject, it is clear that the human immune system can generate a significant neutralizing response against the BoNT catalytic domain. The use of human antibodies specific for the catalytic domains of bacterial toxins may be an important general principle in the development of effective anti-toxins. Finally, the study of these antibodies may provide insights into the way endocytosed proteins are processed by cells, as well as how toxins could be used to deliver drugs or peptides to membranous organelles within a cell.

## Materials and Methods

### Cell culture and 4LCA antibody cloning

Peripheral blood lymphocytes were obtained from a volunteer donor 8 days following a fifth dose of the pentavalent botulinum toxoid vaccine, following a protocol approved by the Institutional Review Board of the Thomas Jefferson University. The peripheral blood mononuclear cell (PBMC) fraction was isolated using gradient density centrifugation with FicollPaque PLUS (GE Healthcare, Piscataway, NJ) and stored under liquid nitrogen in 90% heat-inactivated fetal calf serum (Invitrogen, Carlsbad, CA) 10% DMSO (Sigma-Aldrich, St. Louis, MO). For cell fusion, 1×10^7^ PBMCs were thawed and CD27+ cells were selected using magnetic beads (Miltenyi Biotec, Auburn, CA) and then cultured on a monolayer of irradiated tCD40L cells (courtesy of Gordon Freeman, Dana Farber/Partners Cancer Care, Boston, MA), in IMDM supplemented with 10% human serum, IL-4 (2 ng/ml), IL-10 (10 ng/ml), transferrin (50 µg/ml), cyclosporine A (5.5×10^−4^ M), L-glutamine (2 mM), BoNT/A or HC50A (5 µg/ml) and penicillin/streptomycin (Sigma-Aldrich). After 8 days of culture, 5×10^5^ cells were fused with 5×10^5^ B5-6T cells and plated in 2×96-well plates over a C57BL/6 thymocyte feeder layer. HAT selection was initiated 24 hours after cell fusion and continued for 7 days. Hybridoma pools were screened on day 10 for IgG reactive with the BoNT/A LC and a positive pool was subcloned 3 times to isolate a stable hybridoma expressing the 4LCA antibody. The hybridoma was adapted to serum-free IS MAB-CD (Irvine Scientific, Santa Ana, CA), and cultured for 5 days in a 500 ml roller bottle at an initial density of 5×10^5^ cells/ml in 100 ml culture. Supernatants were filtered and antibodies were purified over a Protein G Sepharose column (GE Healthcare). SDS-PAGE (Invitrogen) and the NanoDrop spectrophotometer (NanoDrop Technologies, Wilmington, DE) were used to assess antibody purity and concentration.

### ELISAs

BoNT/A LC-specific antibodies were identified by ELISA with a recombinant LC, in the form of a recombinant N-terminal domain of BoNT/A (amino acids 1–425), which was produced in *E. coli* and purified following published protocols [28]. EasyWash 96-well plates (Corning) were coated at 4 C overnight with 100 µl/well recombinant BoNT/A LC (at 5 µg/ml) in PBS, washed with PBS/0.05% Tween-20 (Sigma-Aldrich) and then blocked for 1 hour at 37 C with PBS/0.05% Tween-20/5% bovine calf serum/3% goat serum (Sigma-Aldrich). 100 µl hybridoma supernatants were incubated for 2 hours at 37 C, and an HRP-conjugated murine anti-human IgG secondary antibody was used (9040-05, Southern Biotechnology, Birmingham, AL) with OPD as a substrate (Sigma-Aldrich) to detect bound human IgG. The 4LCA lambda light chain and the IgG1 subtype were identified with the HRP-conjugated specific goat polyclonal antibody A5175 (anti-lambda) and the mouse monoclonal I9388 (anti-human IgG1) (both from Sigma-Aldrich).

### RT-PCR amplification of antibody genes

The variable domain sequences of the 4LCA heavy and light chain genes were amplified using RT-PCR with consensus primer sets as described previously [14], [29], [30]. RNA was isolated from hybridoma using RNA Stat 60 (Tel-Test, Inc., Friendswood, TX). Reverse transcriptase reactions were performed with Omniscript RT (Qiagen, Valencia, CA). PCR reactions were performed with Taq (Qiagen, Valencia), for 30 cycles of 94 C, 15 sec; 55 C, 30 sec; 72 C, 60 sec. Amplified sequences were isolated by agarose gel electrophoresis, purified using the QiaQuick Gel Extraction kit (Qiagen), and sequenced by the Kimmel Cancer Center Nucleic Acid Facility, Thomas Jefferson University. DNA sequences were analyzed using the V-Quest program [31].

### Binding measurements

To determine the solution-phase binding affinity of the 4LCA antibody, recombinant BotN/T A LC was titrated into solutions containing a fixed concentration of antibody. All experiments were performed at room temperature. Samples were prepared in Tris-buffered saline (50 mM Tris, 100 mM NaCl, pH 8) supplemented with 1 mM PMSF, 0.02% NaN_3_ and 1 mg/ml BSA. At equilibrium, each solution was passed through the flow cell of a KinExA 3000 flow fluorimeter (Sapidyne Instruments, Boise, ID), and free (unbound) 4LCA mAb was captured using LC covalently coupled to azlactone beads (∼36 µm average size, Pierce Biotechnology, Rockford, IL) [32]. Beads were prepared by incubating 100 mg activated azlactone (Pierce) and 50 mg LC overnight in 1 ml sodium carbonate buffer (50 mM, pH 9) at 4 C, and subsequently blocked by the addition of 150 mg BSA (Sigma-Aldrich). Captured antibody was detected using a rhodamine-labeled, goat-anti-human secondary antibody (Jackson ImmunoResearch, West Grove, PA). The fluorescent signals from multiple titrations were fit to a general bimolecular interaction model (manufacturer's software) that assumes a known concentration of 4LCA mAb, enabling the determination of both an equilibrium dissociation constant (K_D_) and the LC specific activity [33]. For some nonequilibrated 4LCA mAb/LC mixtures, the time course of the association reaction was followed in a similar manner in order to determine an association rate constant (k_on_) [17]. The dissociation rate constant (k_off_) was calculated using the relationship k_off_ = K_D_×k_on_.

### Cell culture-based neutralization assay

*In vitro* neutralization of BoNT by human monoclonal antibodies was assayed with the murine cholinergic neuroblastoma cell line Neuro-2a (CCL-131; ATCC, Manassas, VA). Neuro-2a cells were plated at 5×10^4^ per well in a 24-well tissue culture plate in Opti-MEM with 1% IFS, glutamine and pen/strep (Invitrogen). After 48 hours, the medium was replaced with serum-free Opti-MEM and cells were incubated for an additional 24 hours. 2 µg BoNT/A was incubated with 500 µg antibody in 1 ml serum-free Opti-MEM for 1 hour at room temperature. The mixture was added to the Neuro-2a cells, which were then incubated for 48 hours. Cells were separated from the plates mechanically, boiled for 10 minutes in 1× NuPAGE LDS sample buffer and separated by SDS/PAGE on a 12% Bis-Tris NuPAGE gel in MOPS/SDS running buffer (Invitrogen). Proteins were transferred to 0.2 µm Hybond ECL membrane (GE Healthcare, Piscataway, NJ) and the membrane was soaked in blocking buffer (5% skim milk/PBS) overnight at 4 C. An anti-SNAP-25 mouse monoclonal IgG1 (Santa Cruz Biotechnology, Santa Cruz, CA) was used as the primary antibody, diluted 1∶1000 into blocking buffer and added to blot for 1 hr at room temperature. After washing, the blot was probed for 1 hour with a goat anti-mouse HRP-conjugated secondary antibody (Thermo Scientific, Rockford, IL) at 1∶10,000 dilution and then developed with Super-Signal West Dura Chemiluminescent Substrate (Pierce). Quantitation of the Western blot analysis was done by densitometric analysis with a Kodak imager.

### Fluorescence resonance energy transfer (FRET) assay

The effect of human antibodies on the endopeptidase activity of the BoNT/A LC was measured using fluorescent resonance energy transfer assay with the SNAPtide, a synthetic SNAP-25 cleavage site peptide conjugated to two fluorescent molecules (fluorescein thiocarbamoyl (FITC), and 4-(dimethylaminoazo) benzene-4-carboxyl (DABCYL) [20] (List Biological Laboratories, Inc., Campbell, CA). Cleavage of the peptide by the LC relieves the intramolecular quenching by the FITC and DABCYL moieties. Triplicate reactions of BoNT/A (5 nM) and human monoclonal antibody (30 µM) were mixed in 100 µl reaction volumes, which also contained 20 mM HEPES buffer, pH 8.0, 0.3 mM ZnCl_2_, 1.25 mM DTT and 0.1% Tween-20, pH 8.0. Assay mixtures were incubated for 30 minutes at room temperature, SNAPtide (5 µM) was added, and the mixtures were incubated for two additional hours at 37 C. Samples were transferred to a Corning CoStar black 96-well microtiter plate (Fisher Scientific, Pittsburgh, PA) and mixed with 200 µl EDTA 20 mM. Fluorescence intensity of each sample was measured with a Biotek Synergy-HT spectrophotometer at excitation and emission wavelengths of 485 nm and 523 nm, respectively.

### Confocal microscopy

BoNT/A was labeled with the DyLight 549 microscale antibody labeling kit and the human monoclonal antibodies (6A, 4LCA) were labeled with the DyLight 488 microscale antibody labeling kit (both from Pierce Biotechnology). Labeled BoNT/A (1 µg) was incubated with control medium or monoclonal antibody (500 µg 4LCA or 6A) for one hour at room temperature. Sub-confluent Neuro-2a cells, plated on glass cover slips, were incubated with the BoNT mixtures at 4 C for one hour and then for 2 hours at 37 C. Cells were then washed with PBS for 15 minutes, fixed with 4% paraformaldehyde for 15 mins, and washed again before mounting. Confocal laser scanning was performed on a Olympus Fluoview 500 system using the 40× objective, and Fluoview software was used for image analysis.
